# Telomeric DNA–Promyelocytic Leukemia (TEL–PML) Colocalization as an ALT Proxy in Relation to Metastatic Behavior in Osteosarcoma: A Retrospective Cohort Study

**DOI:** 10.3390/cimb48060553

**Published:** 2026-05-25

**Authors:** Rogelio Frank Jiménez-Ortega, Rosa M. Salgado, Berenice Rivera-Paredez, Nelly Patiño, Tania Hilario-Huerta, Silvia Arenas-Díaz, Rafael Velázquez-Cruz, Alberto Hidalgo-Bravo

**Affiliations:** 1 Servicio de Medicina Genómica, Instituto Nacional de Rehabilitación Luis Guillermo Ibarra Ibarra (INRLGII), Mexico City 14389, Mexico; rfrankjo@gmail.com (R.F.J.-O.); taniahhuerta@hotmail.com (T.H.-H.); sarenas_mel@yahoo.com (S.A.-D.); 2 Laboratorio de Tejido Conjuntivo, Instituto Nacional de Rehabilitación Luis Guillermo Ibarra Ibarra (INRLGII), Mexico City 14389, Mexico; rsalgado@inr.gob.mx; 3 Centro de Investigación en Políticas, Población y Salud, Facultad de Medicina, Universidad Nacional Autónoma de México, Mexico City 04510, Mexico; bereriverap@comunidad.unam.mx; 4 Unidad de Citometría de Flujo (UCiF), Instituto Nacional de Medicina Genómica (INMEGEN), Mexico City 14610, Mexico; lnpatino@inmegen.gob.mx; 5 Laboratorio de Genómica del Metabolismo Óseo, Instituto Nacional de Medicina Genómica (INMEGEN), Mexico City 14610, Mexico; rvelazquez@inmegen.gob.mx

**Keywords:** osteosarcoma, telomere maintenance, alternative lengthening of telomeres, TEL–PML colocalization, TERT immunohistochemistry

## Abstract

Osteosarcoma is the most common primary bone tumor in children, adolescents, and young adults, and metastasis remains the main determinant for a poor outcome. We conducted an exploratory retrospective cohort study using formalin-fixed, paraffin-embedded tissue from 97 patients with histopathologically confirmed osteosarcoma treated between 2005 and 2019 to evaluate telomere maintenance mechanisms. To assess alternative lengthening of telomeres (ALT), colocalization of telomeric DNA and promyelocytic leukemia protein (TEL–PML) was evaluated as a tissue-based proxy. TEL–PML colocalization was assessed using combined PML immunofluorescence and telomere DNA PNA-FISH. Furthermore, telomerase reverse transcriptase (TERT) expression was evaluated by immunohistochemistry in relation to metastasis and disease progression. Logistic regression models were adjusted for age, sex, and smoking. TEL–PML was evaluable in 45/97 cases, including 10 positive and 35 negative tumors; the remaining samples were non-evaluable because of non-determinable signal or predominant necrosis. TERT immunohistochemistry was scorable in 58/97 cases, of which 33 were positive. TEL–PML evaluability was associated with amputation specimens, whereas TERT positivity was associated with non-osteoblastic histology and was inversely associated with age. Neither TEL–PML nor TERT was significantly associated with metastasis, recurrence, or death. Exploratory time-to-metastasis curves suggested an earlier increase of metastatic events among TEL–PML-positive cases. However, the small number of positive tumors precludes definitive prognostic interpretation. These hypothesis-generating findings indicate that TEL–PML assessment is feasible in osteosarcoma, but it is strongly influenced by tissue adequacy. On the other hand, TERT immunohistochemistry appears to reflect subtype- and age-related heterogeneity rather than providing robust outcome stratification in this cohort.

## 1. Introduction

Osteosarcoma (OS) is the most common primary malignant bone tumor. It predominantly affects children, adolescents, and young adults. Despite multidisciplinary treatment, typically combining multi-agent chemotherapy and complete surgical resection, metastatic dissemination remains the major determinant of outcome. Patients with macroscopic metastases, most commonly in the lungs, have substantially poorer survival than those with localized disease. Metastatic behavior remains a major driver of treatment failure in osteosarcoma, including both metastases present at diagnosis and metastatic progression during follow-up [1,2]. Current risk stratification is largely based on clinicopathologic variables, such as metastatic status at diagnosis, histologic response to neoadjuvant chemotherapy, and surgical resection, which are informative but imperfect surrogates of underlying tumor biology. Consequently, there is a persistent need for tissue-based biomarkers that better reflect osteosarcoma biology and may help refine the identification of patients at increased risk of metastatic progression who could benefit from closer follow-up [2].

A biologically compelling and clinically accessible axis for biomarker development is telomere maintenance mechanisms, which enable cancer cell immortality. To achieve replicative immortality, cancer cells counteract telomere erosion, usually by reactivating telomerase. However, a clinically significant subset of tumors uses a recombination-based, telomerase-independent mechanism called alternative lengthening of telomeres (ALT). ALT is enriched in tumors from mesenchymal origin, including sarcomas, and is linked to genomic instability and unique DNA repair dependencies [3,4]. ALT is characterized by ALT-associated promyelocytic leukemia (PML) bodies (APBs), which are nuclear domains where telomeric DNA and telomere repair factors interact with the PML protein. APBs can be identified by telomere FISH combined with PML immunofluorescence and quantified as TEL–PML colocalization. In archival tumor samples, this dual-staining approach provides a practical tissue-based proxy for ALT-associated biology; however, orthogonal assays are required to confirm functional ALT status. Functionally, APBs are not merely passive markers; they promote telomeric recombination and DNA synthesis, which are necessary for ALT-mediated telomere lengthening [5].

Multiple studies indicate that ALT and, by extension, TEL–PML colocalization or APB positivity are common in osteosarcoma, although prevalence estimates vary considerably across reports. Henson et al. identified ALT in 47% of osteosarcomas (27/58) using an APB assay on paraffin sections, Ulaner et al. reported 63% (38/60), and a more recent C-circle assay–based study found ALT in 71% of cases. These differences likely reflect not only biological heterogeneity but also methodological variation, including differences in ALT biomarkers, specimen type, case selection, and the frequent use of a single assay rather than orthogonal confirmation [5,6,7,8]. In sarcomas, ALT is linked to chromatin remodeling and telomere regulation, including the loss of the ATRX protein, although this association is not always present. A large sarcoma series using telomere-specific assays with immunohistochemistry found that ALT features and ATRX deficiency strongly co-occur. This indicates that ATRX loss is often, but not exclusively, associated with ALT, supporting the idea that altered chromatin states promote telomeric recombination and APB formation. In osteosarcoma, recent genomic data further clarify factors associated with ALT, suggesting that genetic pathways beyond ATRX loss can also confer an ALT phenotype [9]. Clinically, the prognostic impact of ALT in sarcomas is inconsistent. Some studies link ALT to worse outcomes in certain subgroups, while others find no effect. This inconsistency likely reflects biological diversity, different endpoint definitions, and methodological differences of ALT detection [10]. A systematic review and meta-analysis highlighted this variability and called for tumor- and context-specific assessments rather than generalizing across all sarcomas [11]. Given the central role of metastatic behavior in osteosarcoma mortality, the feasibility of assessing APBs on archival samples, and the biological plausibility that APB-positive tumors may reflect ALT-associated telomere biology, we performed an exploratory retrospective study to evaluate APB presence as an ALT proxy, related to metastatic behavior in osteosarcoma, including hypothesis-generating analyses of time-to-metastasis patterns [5,12].

## 2. Materials and Methods

### 2.1. Study Population

A retrospective, observational cohort study was conducted in patients with a histopathological diagnosis of osteosarcoma who were treated at the National Institute of Rehabilitation “Luis Guillermo Ibarra Ibarra” (INR) between November 2005 and November 2019. The study was approved by the institutional research and ethics committees (approval number 29-15). For retrospective review of archived tissue specimens and clinical records, patient consent was waived with ethics committee approval. Tumor tissue samples were obtained from diagnostic biopsies performed for tumor confirmation and classification, as well as from surgical resection specimens from patients treated at the INR, including those referred from other institutions and/or previously treated with chemotherapy. Healthy bone from tumor-free surgical margins (“clean margins”), with no evidence of malignant activity, confirmed by histopathological analysis, was used as control tissue. Patients were followed through available clinical records to document relevant clinical outcomes, including response to chemotherapy, metastasis, relapse, and death. For time-to-metastasis analyses, follow-up was administratively censored at 36 months, corresponding to the maximum recorded Time-SX–METS in the dataset.

### 2.2. Data Curation and Analytical Dataset

After data extraction from clinical records, pathology reports, and biomarker assessment forms, the study database was curated before analysis. Duplicate records were identified using the unique Folio identifier and removed when present, retaining only one record per patient. Variable names were standardized, blank entries were harmonized as missing values, and range and consistency checks were performed for continuous and categorical variables before statistical analysis. For outcome analyses, metastasis was evaluated using two complementary approaches. First, metastatic status (METS) was analyzed as a binary endpoint in all cases with available information. Second, time-to-metastasis analyses were performed in the subset of patients with sufficient information to define a surgery-based time origin and event/censoring status. A recorded Time_SX–METS value was available only for patients who developed metastasis and had a documented surgery-to-metastasis interval, whereas patients without metastasis could still contribute censored observations when surgery-based follow-up information was available. Vital status, relapse, and chemotherapy-related variables were analyzed according to the availability of complete-case data for each model. TEL–PML colocalization was recorded for all available samples and classified as positive, negative, predominant necrosis, or non-determinable (ND) according to signal quality and interpretability. For analyses of APB positivity, only evaluable cases (positive or negative) were included, whereas ND and predominant necrosis were grouped as non-evaluable when evaluability itself was modeled as an outcome. TERT immunohistochemistry was analyzed only in scorable samples.

### 2.3. APBs Determination (TEL–PML Colocalization, PML Immunofluorescence + Telomere PNA–FISH)

Formalin-fixed, paraffin-embedded (FFPE) osteosarcoma tissue sections (from diagnostic biopsies or surgical resections) were analyzed using a combined immunofluorescence–FISH assay to detect ALT-associated PML bodies (APBs). Briefly, sections were immunostained with a mouse anti-PML primary antibody diluted 1:50 in TBS with albumin, followed by an Alexa Fluor 488-conjugated anti-mouse IgG secondary antibody (Thermo Fisher Scientific, Inc., Waltham, MA, USA) diluted 1:50. Telomeric DNA was subsequently detected by fluorescence in situ hybridization using a telomere-specific fluorescent PNA probe (CCCTAACCCTAACCCTAA) with the N-terminal covalently linked to Cy3 (PNA Bio, Inc., Newbury Park, CA, USA). Nuclei were counterstained with DAPI, and the slides were evaluated using an Axio Observer Z1 microscope (Carl Zeiss Microscopy GmbH, Jena, Germany). APB status was assessed by the research team and classified as positive, negative, predominant necrosis, or non-determinable (ND) according to signal quality and interpretability. Only evaluable cases (positive/negative) were included in analyses of APB positivity. Throughout the manuscript, APB positivity refers to TEL–PML colocalization and is interpreted as an ALT proxy rather than definitive functional confirmation of ALT status. Because this was a retrospective FFPE-based cohort with limited residual archival material, orthogonal ALT assays, including C-circle testing and ATRX immunohistochemistry, were not available for the present analysis. Therefore, TEL–PML positivity should be considered an APB-based surrogate marker of ALT-associated telomere maintenance rather than definitive evidence of functional ALT activation.

### 2.4. Immunohistochemistry

Immunohistochemistry was performed on 5-µm sections from FFPE osteosarcoma tissue. Sections were deparaffinized in xylene and rehydrated through graded alcohols. Antigen retrieval was carried out in citrate buffer (pH 6.0) by heating the slides on a hot plate at 75 °C for 15 min, followed by cooling at room temperature for 15 min. Slides were then washed with PBS and incubated with a blocking solution (Vectastain Universal Quick HRP Kit, Peroxidase R.T.U.; Vector Laboratories, Inc., Newark, CA, USA) for 10 min. After removing excess reagent, sections were incubated with a primary monoclonal antibody against human TERT (A-6; Santa Cruz Biotechnology, Inc., Dallas, TX, USA) at 1:50 dilution for 18 h at 4 °C. Immunoreactivity was developed using an AEC system (AEC Substrate Kit, Peroxidase [HRP]; Vector Laboratories, Inc., Newark, CA, USA), counterstained with Harris hematoxylin, mounted with Vectamount AQ (Vector Laboratories, Inc., Newark, CA, USA), and examined using an Axio Observer Z1 microscope (Carl Zeiss Microscopy GmbH, Jena, Germany). Semi-quantitative assessment of TERT staining was based on the percentage of positive cells and staining intensity, using a scoring system modified from Jayaraj et al. (2022); scoring was performed concurrently by one pathologist and two observers using a multihead microscope, and a consensus score was assigned for each slide [13].

### 2.5. Statistical Analysis

Analyses were conducted in R v4.5.2. Continuous variables were summarized as mean ± SD or median (IQR), and categorical variables as *n* (%). Associations between telomere-related biomarkers and clinical variables were evaluated using logistic regression. APBs (derived from TEL–PML colocalization) were analyzed using two definitions: evaluable versus non-evaluable (ND/predominant necrosis), and APB positivity among evaluable cases only (TEL–PML positive vs. TEL–PML negative). Each clinicopathological predictor was examined in a separate logistic regression model adjusted a priori for age, sex, and smoking, rather than in a single fully saturated model, to reduce overfitting given the limited sample size and number of outcome events; when age was the predictor, models were adjusted for sex and smoking only. Because predictors were not entered simultaneously into a single multivariable model, multicollinearity among candidate clinicopathologic variables was not expected to materially affect the main analyses. Sparse categories were collapsed when needed. To improve clinical interpretability, logistic regression results were reported primarily as odds ratios (ORs) with 95% confidence intervals and two-sided *p* values, whereas regression coefficients (β) were treated as underlying model parameters rather than the main effect measure. N reflected complete-case data for each model. Because TEL–PML status was non-evaluable in a substantial subset of specimens, TEL–PML-evaluable and non-evaluable cases were compared using the Mann–Whitney U test for continuous variables and Fisher’s exact test for categorical variables. As a sensitivity analysis focused on potential treatment-related confounding, the association between TEL–PML evaluability and the main predictor of interest was re-estimated in separate logistic regression models by adding treatment-related variables available in the database, including initial treatment category, neoadjuvant chemotherapy, adjuvant chemotherapy, and radiotherapy. These analyses were performed using complete-case data for each model and were intended to assess the robustness of the primary age-, sex-, and smoking-adjusted estimates. To further reduce small-sample and sparse-data bias, the same treatment-adjusted sensitivity models were additionally fitted using Firth penalized logistic regression. In addition, because TEL–PML positivity was observed in only 10 evaluable tumors, univariable Firth penalized logistic regression models were fitted as exploratory sensitivity analyses to evaluate the association between TEL–PML positivity and clinical outcomes among evaluable cases. TEL–PML positivity was used as the predictor of interest, and the outcomes included metastasis during follow-up, recurrence, death at last follow-up, and early metastasis among cases with recorded timing data. Given the limited number of TEL–PML-positive cases and outcome events, these models were not further adjusted for covariates and were interpreted descriptively.

To further address potential bias related to TEL–PML non-evaluability, inverse probability weighting (IPW) was performed as an additional sensitivity analysis. First, the probability of TEL–PML evaluability was estimated in the full cohort using a logistic regression model that included age, sex, smoking, amputation specimen, and non-osteoblastic histologic subtype; missing-indicator terms were used for variables with incomplete data. Stabilized inverse probability weights were then calculated and applied to TEL–PML-evaluable cases. IPW-weighted logistic regression models were used to evaluate the association between TEL–PML positivity and clinical outcomes, including metastasis during follow-up, recurrence, death at last follow-up, and early metastasis among cases with recorded timing data. These models were interpreted as exploratory sensitivity analyses for evaluability-related missingness.

Variables such as specific chemotherapy regimen, histologic response to treatment, and initial tumor stage were considered clinically relevant a priori, but they were not incorporated because they were not consistently available in the curated dataset. *p*-values were interpreted descriptively (nominal *p* < 0.05).

### 2.6. Time-to-Metastasis Analysis

Kaplan–Meier methods were used to evaluate time to metastasis, and results were presented as the cumulative probability of metastasis over time, estimated as 1−Kaplan–Meier. Time-to-event was defined from surgery to documented metastasis (Time_SX–METS) for patients with metastatic events and a recorded interval. Patients without metastasis contributed censored observations when sufficient information was available to define the surgery-based risk period. When no follow-up time was recorded, administrative censoring was set at 36 months, corresponding to the maximum observed Time_SX–METS in the dataset. Accordingly, the Kaplan–Meier analysis was based on a surgery-based analytical subset and not on the full cohort count with recorded Time_SX–METS values alone. Curves were stratified by APB status as an ALT proxy; non-evaluable specimens (ND or predominant necrosis) formed a separate category. As the time to death was unavailable, death counts per group were reported in the figure legend.

Because exact time-to-death information was not available, formal competing-risk analyses using death as a competing event could not be validly implemented. Therefore, time-to-metastasis curves were retained only as descriptive, hypothesis-generating visualizations rather than inferential competing-risk estimates. Radiobiological modeling was not performed because detailed radiotherapy parameters, including dose, fractionation, treatment fields, target volumes, timing relative to surgery and chemotherapy, and local-control endpoints, were not consistently available. In addition, the limited number of patients who received radiotherapy precluded stable model estimation.

## 3. Results

### 3.1. Cohort Characteristics and Biomarker Evaluability

In total, 97 patients with histopathologically confirmed osteosarcoma treated at the INR between November 2005 and November 2019 were included after data curation. The cohort had a median age at diagnosis of 19 years (IQR 15–28; range 7–66) and comprised 51 males (52.60%) and 46 females (47.40%). Data completeness was high for the main clinical outcomes, with metastasis available for 94/97 cases and vital status for 95/97. A recorded Time_SX–METS value was available for 54/97 cases, corresponding to patients with documented metastatic events and an available surgery-to-metastasis interval. On the other hand, time-to-event analyses additionally included censored patients within a separate surgery-based analytical subset. Metastasis was documented for 66/94 patients (70.2%), recurrence for 11/97 (11.3%), and death for 62/95 (65.3%). TEL–PML colocalization was available for all cases, but only 45/97 (46.4%) were evaluable, including 10/45 (22.2%) TEL–PML-positive and 35/45 (77.8%) TEL–PML-negative tumors; the remaining samples were classified as non-determinable (22/97, 22.7%) or as showing predominant necrosis (30/97, 30.9%). Therefore, TEL–PML positivity was interpreted only among evaluable specimens and was not considered an unbiased cohort-level estimate of ALT-proxy prevalence.

To assess potential sources of technical evaluability bias, TEL–PML-evaluable and non-evaluable specimens were compared. The main difference between groups was a higher proportion of amputation specimens among TEL–PML-evaluable cases, whereas age, sex, smoking, treatment-related variables, metastasis, recurrence, and death did not differ significantly between evaluable and non-evaluable specimens (Supplementary Table S1). Among the evaluable TEL–PML cases, clinicopathologic characteristics according to TEL–PML status are summarized in Table 1.

TERT immunohistochemistry was scorable for 58/97 samples (59.8%); from these, 33/58 (56.9%) were TERT-positive (score ≥ 1) and 25/58 (43.1%) were TERT-negative (score 0), with score distribution as follows: score 0 (*n* = 25), score 1 (*n* = 13), score 2 (*n* = 6), and score 3 (*n* = 14). The distribution of TERT immunohistochemistry across clinicopathologic characteristics is summarized in Table 2.

**Figure 1:**
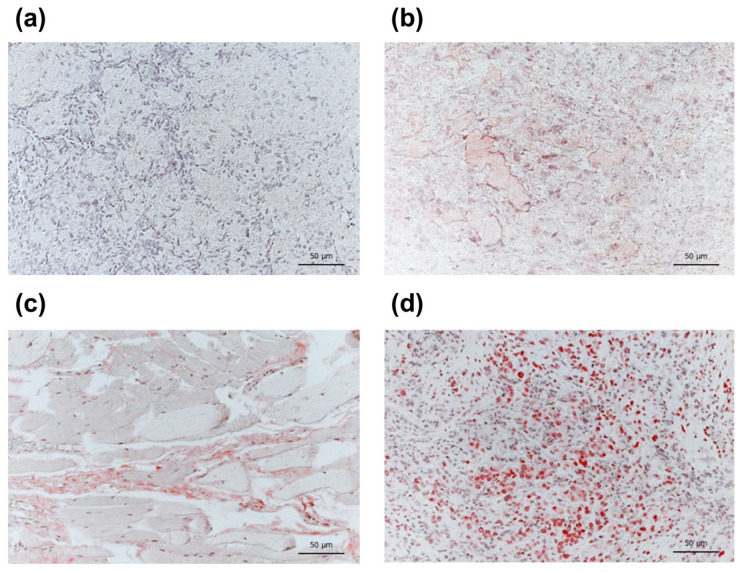
TERT expression by immunohistochemistry in osteosarcoma. Representative 20× photomicrographs of FFPE sections stained with anti-TERT and developed with AEC. (**a**) Score 0: <10% positive tumor cells (negative). (**b**) Score 1: 10–30% (weak expression). (**c**) Score 2: 31–50% (moderate expression). (**d**) Score 3: >50% (strong/intense expression). Red staining indicates positive TERT immunoreactivity (AEC signal), while blue-purple nuclei correspond to hematoxylin counterstaining.

On FFPE osteosarcoma sections stained with anti-TERT, immunoreactivity was visualized as a red AEC signal and showed heterogeneous distribution across cases. Hematoxylin counterstaining supported morphologic assessment and tumor cell enumeration. TERT expression was classified using a semi-quantitative scoring system modified from Jayaraj et al. (2022) [13], ranging from score 0 (<10% positive tumor cells; negative staining) to score 3 (>50% positive tumor cells; strong staining). Negative samples showed absent or minimal staining (<10% positive tumor cells), whereas scores 1–3 reflected increasing proportions and intensity of immunoreactive tumor cells (score 1 = 10–30%, score 2 = 31–50%, and score 3 ≥ 50%). Representative staining patterns for each category are shown in Figure 1.

### 3.2. Clinicopathologic Correlates of TEL–PML and TERT

Histologic subtype was first explored descriptively using the grouped categories shown in Table 2. For adjusted logistic regression, however, this variable was collapsed into osteoblastic versus non-osteoblastic subtype to reduce sparse-cell counts and improve model stability. Therefore, the reported association reflects this binary contrast rather than the evaluation of a single individual subtype. In separate parsimonious logistic regression models adjusted a priori for age, sex, and smoking, TEL–PML evaluability (evaluable vs. non-evaluable) was associated with amputation (OR 2.99, 95% CI 1.18–7.52; *p* = 0.020). TEL–PML positivity among evaluable cases showed a borderline association with an initial presentation of pain and mass/volume increase (OR 4.67, 95% CI 0.87–25.2; *p* = 0.073). TERT positivity (IHC score ≥ 1 vs. 0) was associated with non-osteoblastic histologic subtype (OR 4.58, 95% CI 1.32–15.9; *p* = 0.016) and inversely associated with age at diagnosis (OR per year 0.95, 95% CI 0.91–0.99; *p* = 0.027). These results are summarized in Table 3.

### 3.3. Treatment-Adjusted Sensitivity Analyses

In treatment-adjusted sensitivity analyses, the association between amputation and higher TEL–PML evaluability remained consistent after adjustment for age, sex, smoking, and initial treatment category, neoadjuvant chemotherapy, or adjuvant chemotherapy. The radiotherapy-adjusted model showed the same direction of association but did not reach statistical significance, likely due to the smaller complete-case sample (Supplementary Table S2). To further address sparse-data bias, the same sensitivity analyses were repeated using Firth penalized logistic regression. These models confirmed higher odds of TEL–PML evaluability among amputation specimens after adjustment for initial treatment category, neoadjuvant chemotherapy, or adjuvant chemotherapy, with a similar but non-significant trend in the radiotherapy-adjusted model (Supplementary Table S3). Overall, these findings support the robustness of the primary result to the treatment-related variables available in the dataset and indicate that TEL–PML evaluability was partly related to specimen characteristics. Remaining non-significant associations are summarized in Supplementary Table S4.

### 3.4. Descriptive Exploratory Time-to-Metastasis Analysis

**Figure 2:**
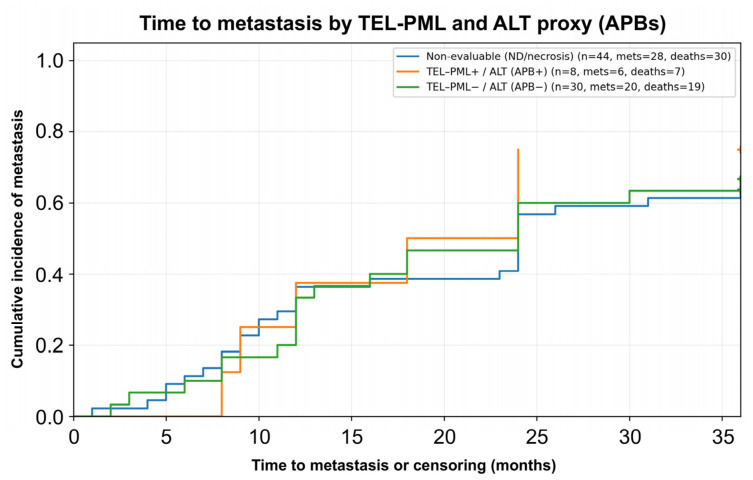
Time to metastasis according to TEL–PML status and APB-based ALT proxy. Descriptive cumulative probability of metastasis was estimated as 1 − Kaplan–Meier and plotted from surgery to metastasis or censoring (months). Curves are shown for non-evaluable TEL–PML specimens (ND/predominant necrosis; *n* = 44, metastases = 28, deaths = 30), TEL–PML-positive/APB-positive tumors interpreted here as an ALT proxy (*n* = 8, metastases = 6, deaths = 7), and TEL–PML-negative/APB-negative tumors (*n* = 30, metastases = 20, deaths = 19). The APB-positive group showed an earlier increase in metastatic events, whereas the APB-negative group displayed a more gradual rise; however, the number of APB-positive cases was small, precluding definitive prognostic interpretation. Note: This Kaplan–Meier analysis was performed in the subset of patients with sufficient information to define surgery-based event/censoring times. Therefore, the denominators shown in this figure differ from the full-cohort TEL–PML counts and from the 54 cases with a recorded Time_SX–METS value, which refers only to patients with documented metastatic events and an available surgery-to-metastasis interval.

In time-to-event analyses, Figure 2 was based on the subset of patients with sufficient information to define a surgery-based event/censoring framework, which explains why the denominators in the Kaplan–Meier analysis differ from the total number of cohort members with a recorded Time_SX–METS value and from the overall TEL–PML classification counts. Within this analytical subset, 1−Kaplan–Meier curves showed a high metastatic burden among evaluable TEL–PML categories. The APB-positive group, used here as an ALT proxy, showed an early increase in metastasis probability (6 events among 8 patients), whereas the APB-negative group showed a more gradual rise (20 events among 30 patients). Consistent with this pattern, the overall frequency of metastasis among evaluable cases included in the time-to-event subset was similar between TEL–PML-positive and TEL–PML-negative groups (75.0% vs. 70.6%) (Figure 2; Supplementary Table S5). To further address sparse-data bias related to the low number of TEL–PML-positive/APB-positive tumors, univariable Firth penalized logistic regression models were performed among TEL–PML-evaluable cases. TEL–PML positivity was not robustly associated with metastasis during follow-up (Firth OR 1.11, 95% CI 0.23–6.86; *p* = 0.896), recurrence (Firth OR 1.11, 95% CI 0.10–7.02; *p* = 0.922), death at last follow-up (Firth OR 1.80, 95% CI 0.40–10.75; *p* = 0.454), or early metastasis among cases with recorded timing data (Firth OR 0.38, 95% CI 0.003–4.85; *p* = 0.507) (Supplementary Table S6). These results support the interpretation that outcome analyses according to TEL–PML positivity remain exploratory and underpowered. As an additional sensitivity analysis for evaluability-related missingness, IPW-weighted logistic regression models were fitted among TEL–PML-evaluable cases. After weighting by the inverse probability of TEL–PML evaluability, TEL–PML positivity was not robustly associated with metastasis during follow-up (IPW-weighted OR 1.37, 95% CI 0.19–9.84; *p* = 0.755), recurrence (IPW-weighted OR 0.74, 95% CI 0.04–12.90; *p* = 0.837), or death at last follow-up (IPW-weighted OR 1.85, 95% CI 0.27–12.75; *p* = 0.534). Early metastasis was not estimable in the IPW-weighted model because no early metastatic events occurred among TEL–PML-positive cases with recorded timing data (Supplementary Table S7). Clinical outcomes according to TERT immunohistochemistry status are summarized in Supplementary Table S8.

## 4. Discussion

This exploratory retrospective study evaluated two tissue-based markers related to telomere maintenance mechanisms in archival osteosarcoma: TEL–PML colocalization as an ALT proxy and TERT immunohistochemistry, in relation to metastatic behavior and clinicopathologic features. The main findings were that TEL–PML evaluability depended strongly on specimen adequacy, and this association remained consistent after treatment-adjusted sensitivity analyses. In addition, we observed that TERT positivity varied according to tumor histologic subtype and age. By contrast, neither TEL–PML nor TERT was significantly associated with metastasis, recurrence, or death. Together, these findings suggest that, in retrospective FFPE material, assay interpretability and pre-analytical constraints may be as important as tumor biology when telomere-related biomarkers are assessed in osteosarcoma [14,15,16,17]. The relatively low proportion of APB-positive cases among evaluable samples should therefore be interpreted cautiously, particularly because larger or more tissue-rich specimens, such as amputation samples, were more likely to yield interpretable TEL–PML results. This supports a specimen-level explanation in which tissue adequacy materially conditions biomarker assessment in archival samples [14,18]. Importantly, the high proportion of non-evaluable specimens, particularly those with predominant necrosis, may have introduced selection bias in the representation of telomere-maintenance mechanisms. Because evaluable TEL–PML results were more frequently obtained from larger or more tissue-rich specimens, including amputation samples, APB positivity should not be interpreted as the true prevalence of ALT-proxy biology in the entire cohort. Rather, TEL–PML findings should be interpreted as biomarker results conditional on tissue interpretability.

The biological interpretation of TEL–PML and TERT findings also requires caution. Prior studies have shown substantial heterogeneity in ALT prevalence across osteosarcoma and other sarcomas, and different ALT assays do not necessarily identify the same subset of tumors [5,6,7,8,9,18,19,20]. In our cohort, 1−Kaplan–Meier curves suggested an earlier rise in metastatic events among APB-positive cases, but the number of evaluable positive tumors was small, and confidence in this pattern is limited. Consistently, exploratory Firth penalized logistic regression models did not identify robust associations between TEL–PML positivity and metastasis, recurrence, death, or early metastasis, reinforcing that these clinical outcome analyses should be considered hypothesis-generating rather than confirmatory.

Similarly, IPW-weighted sensitivity analyses accounting for TEL–PML evaluability-related missingness did not materially change these findings, further supporting the absence of a robust clinical association in this dataset. Likewise, TERT positivity was associated with non-osteoblastic subtype and younger age, but not with adverse outcomes. This differs from previous osteosarcoma studies in which telomerase activity or telomerase expression was linked to poorer prognosis or was more frequently detected in metastatic lesions than in primary tumors [10,21,22,23,24]. One likely explanation is methodological: semiquantitative immunohistochemistry may capture a different dimension of tumor heterogeneity than functional telomerase assays or transcript-based measurements. The borderline association between TEL–PML positivity and pain plus mass/volume increase should also be considered hypothesis-generating rather than conclusive. A major limitation of this study is that the available sample size differed according to the biomarker or endpoint evaluated, with TEL–PML evaluable in 45 cases, TERT scorable in 58, and Time_SX–METS available in 54 metastatic cases.

This introduces potential selection bias because patients with interpretable tissue or complete timing data may differ systematically from those without such information. In exploratory comparisons, TEL–PML-evaluable cases were more often amputation specimens than non-evaluable cases (Supplementary Table S1). TERT-scorable cases were also enriched for amputation specimens and deaths at last follow-up, and among metastatic patients, recorded Time_SX–METS was more common in those who died than in those who remained alive. These patterns suggest that missingness was not fully random and may have affected both precision and generalizability [15,16]. In addition, a formal competing-risk analysis for time to metastasis could not be performed because exact time-to-death information was unavailable. For this reason, time-to-metastasis curves were interpreted descriptively, with death counts reported by the TEL–PML group, rather than as definitive cumulative incidence estimates. Additional limitations include the retrospective single-center design, the small number of APB-positive cases, the lack of orthogonal functional ALT confirmation, and the absence of some clinically relevant covariates such as chemotherapy regimen, histologic response, and initial tumor stage. Although TEL–PML colocalization supports the presence of APB-like ALT-associated nuclear structures, it does not provide functional confirmation of ALT activity. Orthogonal assays such as C-circle testing, ATRX immunohistochemistry, or telomere length profiling were not available in this retrospective FFPE cohort. Therefore, TEL–PML findings should be interpreted as exploratory APB-based ALT-proxy results rather than definitive evidence of functional ALT activation. In addition, radiobiological modeling could not be performed because detailed radiotherapy parameters and local-control endpoints were not consistently available, and the number of irradiated cases was limited. Future prospective studies should integrate TEL–PML/APB detection with C-circle assays, ATRX status, detailed telomere profiling, and complete radiotherapy data to evaluate whether ALT-associated mechanisms influence treatment response and clinical outcomes in osteosarcoma. Despite this, the study has important strengths, including blinded TEL–PML assessment, evaluation of two complementary telomere-maintenance surrogates, and explicit distinction between biomarker positivity and sample evaluability. Overall, our findings suggest that TEL–PML assessment in osteosarcoma is feasible but highly dependent on tissue adequacy, whereas TERT immunohistochemistry captures subtype- and age-related heterogeneity without supporting robust outcome stratification in this dataset. Future prospective studies should prioritize standardized pretreatment sampling and orthogonal ALT/telomerase assays to better define the clinical value of telomere-maintenance biomarkers in osteosarcoma.

## 5. Conclusions

In this retrospective osteosarcoma cohort, TEL–PML colocalization was feasible as a tissue-based proxy for alternative lengthening of telomeres, although its assessment was strongly influenced by specimen adequacy and tissue interpretability. TERT immunohistochemistry captured heterogeneity related to histologic subtype and age at diagnosis, but neither TEL–PML nor TERT showed a robust association with metastasis, recurrence, or death in this dataset. Although exploratory time-to-metastasis analyses suggested an earlier rise in metastatic events among TEL–PML-positive/APB-positive cases, the limited number of evaluable positive tumors precludes firm prognostic inference. Overall, these hypothesis-generating findings support the technical relevance of telomere-maintenance biomarkers in osteosarcoma while highlighting the importance of sample quality, larger multicenter prospective cohorts with increased numbers of TEL–PML-positive/APB-positive cases, and orthogonal ALT and telomerase assays to better define their clinical value. Such studies should also include complete radiotherapy parameters to enable radiobiological modeling of telomere-maintenance biomarkers in relation to treatment response and clinical outcomes.

## Figures and Tables

**Table 1:** TEL–PML colocalization and association with clinicopathologic characteristics among evaluable cases.

Parameter	Level	TEL–PML (+) (*n* = 10)	TEL–PML (−) (*n* = 35)	*p*-Value
Age at diagnosis (years)	≥20 (*n* = 23)	3 (13.0%)	20 (87.0%)	0.165
	<20 (*n* = 22)	7 (31.9%)	15 (68.2%)	
Sex	Female (*n* = 18)	3 (16.7%)	15 (83.3%)	0.716
	Male (*n* = 27)	7 (26.0%)	20 (74.1%)	
Anatomic region	Lower extremity (*n* = 33)	8 (24.2%)	25 (76.8%)	0.525
	Upper extremity (*n* = 9)	1 (11.1%)	8 (89.0%)	
	Pelvis/iliac (*n* = 2)	0 (0.0%)	2 (100.0%)	
Histologic subtype (grouped)	Osteoblastic (*n* = 17)	5 (29.4%)	12 (70.6%)	0.343
	Osteoblastic/Chondroblastic (*n* = 9)	1 (11.1%)	8 (89.0%)	
	Telangiectatic (*n* = 8)	3 (37.5%)	5 (62.5%)	
	Other (*n* = 11)	1 (9.1%)	10 (91.0%)	
Initial treatment	Chemotherapy (*n* = 26)	5 (19.2%)	21 (80.8%)	0.748
	Surgery (*n* = 10)	3 (30.0%)	7 (70.0%)	
	Chemotherapy/Surgery (*n* = 2)	0 (0.0%)	2 (100.0%)	
	ND (*n* = 7)	2 (28.6%)	5 (71.4%)	
Neoadjuvant chemotherapy	Yes (*n* = 30)	5 (16.7%)	25 (83.3%)	0.217
	No (*n* = 11)	4 (36.4%)	7 (63.6%)	
Adjuvant chemotherapy	Yes (*n* = 37)	8 (21.6%)	29 (78.4%)	1.000
	No (*n* = 3)	0 (0.0%)	3 (100.0%)	
Radiotherapy	Yes (*n* = 1)	0 (0.0%)	1 (100.0%)	1.000
	No (*n* = 20)	6 (30.0%)	14 (70.0%)	
Amputation	Yes (*n* = 28)	7 (25.0%)	21 (75.0%)	0.399
	No (*n* = 11)	1 (9.1%)	10 (90.9%)	
Metastasis during follow-up	Yes (*n* = 30)	6 (20.0%)	24 (80.0%)	1.000
	No (*n* = 12)	2 (16.7%)	10 (83.3%)	
Recurrence	Yes (*n* = 5)	1 (20.0%)	4 (80.0%)	1.000
	No (*n* = 40)	9 (22.5%)	31 (77.5%)	
Vital status at last follow-up	Deceased (*n* = 29)	7 (24.1%)	22 (75.9%)	0.695
	Alive (*n* = 15)	2 (13.3%)	13 (86.7%)	
Smoking	Yes (*n* = 6)	1 (16.7%)	5 (83.3%)	1.000
	No (*n* = 38)	8 (21.1%)	30 (79.0%)	
Time from diagnosis to treatment (months)	Median (IQR)	3.0 (2.0–5.0)	2.0 (1.0–3.0)	0.226

**Table 2:** TERT immunohistochemical expression and association with clinicopathologic characteristics.

Parameter	Level	TERT (+) (*n* = 33)	TERT (−) (*n* = 25)	*p*-Value
Age at diagnosis (years)	≥20 (*n* = 29)	13 (44.8%)	16 (55.2%)	0.110
	<20 (*n* = 29)	20 (69.0%)	9 (31.0%)	
Sex	Female (*n* = 27)	13 (48.2%)	14 (51.9%)	0.289
	Male (*n* = 31)	20 (64.5%)	11 (35.5%)	
Anatomic region	Lower extremity (*n* = 45)	27 (60.0%)	18 (40.0%)	0.823
	Upper extremity (*n* = 10)	5 (50.0%)	5 (50.0%)	
	Pelvis/iliac (*n* = 2)	1 (50.0%)	1 (50.0%)	
Histologic subtype (grouped)	Osteoblastic (*n* = 21)	7 (33.3%)	14 (66.7%)	0.031
	Osteoblastic/Chondroblastic (*n* = 13)	9 (69.2%)	4 (30.8%)	
	Telangiectatic (*n* = 9)	5 (55.6%)	4 (44.4%)	
	Other (*n* = 15)	12 (80.0%)	3 (20.0%)	
Initial treatment	Chemotherapy (*n* = 32)	22 (68.8%)	10 (31.3%)	0.146
	Surgery (*n* = 16)	7 (43.8%)	9 (56.3%)	
	Chemotherapy/Surgery (*n* = 3)	2 (66.7%)	1 (33.3%)	
	ND (*n* = 7)	2 (28.6%)	5 (71.4%)	
Neoadjuvant chemotherapy	Yes (*n* = 38)	25 (65.8%)	13 (34.2%)	0.123
	No (*n* = 15)	6 (40.0%)	9 (60.0%)	
Adjuvant chemotherapy	Yes (*n* = 48)	30 (62.5%)	18 (37.5%)	0.147
	No (*n* = 5)	1 (20.0%)	4 (80.0%)	
Radiotherapy	Yes (*n* = 4)	2 (50.0%)	2 (50.0%)	1.000
	No (*n* = 24)	13 (54.2%)	11 (45.8%)	
Amputation	Yes (*n* = 35)	19 (54.3%)	16 (45.7%)	0.772
	No (*n* = 18)	11 (61.1%)	7 (38.9%)	
Metastasis during follow-up	Yes (*n* = 42)	24 (57.1%)	18 (42.9%)	1.000
	No (*n* = 13)	7 (53.9%)	6 (46.2%)	
Recurrence	Yes (*n* = 9)	4 (44.4%)	5 (55.6%)	0.478
	No (*n* = 49)	29 (59.2%)	20 (40.8%)	
Vital status at last follow-up	Deceased (*n* = 42)	22 (52.4%)	20 (47.6%)	0.226
	Alive (*n* = 15)	11 (73.3%)	4 (26.7%)	
Smoking	Yes (*n* = 5)	3 (60.0%)	2 (40.0%)	1.000
	No (*n* = 52)	30 (57.7%)	22 (42.3%)	
Time from diagnosis to treatment (months)	Median (IQR)	2.0 (1.0–3.0)	2.0 (1.0–4.0)	0.528

TERT (+) was defined as an immunohistochemistry score ≥ 1, and TERT (−) as a score of 0. Analyses were restricted to cases with scorable TERT immunohistochemistry (*n* = 58). Percentages are presented row-wise within each category level. *p*-values were calculated using Fisher’s exact test for 2 × 2 comparisons, Pearson’s chi-square test for larger categorical comparisons, and the Mann–Whitney U test for continuous variables. Age at diagnosis was categorized as <20 versus ≥20 years.

**Table 3:** Separate covariate-adjusted logistic regression models for TEL–PML and TERT outcomes.

Outcome	Predictor	N	OR	OR 95% CI	*p*-Value
TEL–PML evaluable (vs. non-evaluable)	Amputation (yes vs. no)	86	2.99	1.18–7.52	0.020
TEL–PML positivity among evaluable cases only (positive vs. negative)	Initial symptom: pain + mass/volume increase (vs. other symptom patterns)	43	4.67	0.87–25.2	0.073
TERT positive (IHC score ≥1 vs. 0)	Non-osteoblastic (vs. osteoblastic) subtype	57	4.58	1.32–15.9	0.016
TERT positive (IHC score ≥1 vs. 0)	Age (per year)	57	0.95	0.91–0.99	0.027

TEL–PML evaluability was defined as positive or negative versus non-evaluable (non-determinable [ND] or predominant necrosis). TEL–PML positivity analyses were restricted to evaluable cases only and compared TEL–PML-positive versus TEL–PML-negative tumors. TERT positivity was defined as an IHC score ≥ 1 versus a score of 0. The non-osteoblastic subtype was defined as any subtype other than osteoblastic. The initial symptom variable “pain + mass/volume increase” was derived from Sx_inic. Each estimate corresponds to a separate logistic regression model including one predictor of interest plus age, sex, and smoking; results are not derived from a single fully saturated multivariable model. N reflects complete-case data for each model. IHC, immunohistochemistry; OR, odds ratio; CI, confidence interval.

## Data Availability

The data presented in this study are not publicly available due to ethical and privacy restrictions involving human clinical data and archived tissue specimens.

## Supplementary Materials

The following supporting information can be downloaded at: https://www.mdpi.com/article/10.3390/cimb48060553/s1.

## Abbreviations

The following abbreviations are used in this manuscript:

AEC	3-Amino-9-ethylcarbazole
ALT	Alternative lengthening of telomeres
APBs	ALT-associated PML bodies
CI	Confidence interval
DAPI	4′,6-Diamidino-2-phenylindole
DNA	Deoxyribonucleic acid
FFPE	Formalin-fixed, paraffin-embedded
FISH	Fluorescence in situ hybridization
HRP	Horseradish peroxidase
IHC	Immunohistochemistry
INR	Instituto Nacional de Rehabilitación
INRLGII	Instituto Nacional de Rehabilitación “Luis Guillermo Ibarra Ibarra”
IQR	Interquartile range
METS	Metastasis
ND	Non-determinable
OR	Odds ratio
OS	Osteosarcoma
PBS	Phosphate-buffered saline
PML	Promyelocytic leukemia
PNA	Peptide nucleic acid
SD	Standard deviation
TBS	Tris-buffered saline
TEL–PML	Telomeric DNA–promyelocytic leukemia colocalization
TERT	Telomerase reverse transcriptase
