# Supplementary material for: Telomeric DNA–Promyelocytic Leukemia (TEL–PML) Colocalization as an ALT Proxy in Relation to Metastatic Behavior in Osteosarcoma: A Retrospective Cohort Study
Source: Curr Issues Mol Biol. 2026 May 25;48(6):553. doi: 10.3390/cimb48060553 (PMC13297514; doi:10.3390/cimb48060553)
Supplement: Supplementary file 1 [file cimb-48-00553-s001.zip › Table S1.pdf]

Table S1. Comparison of TEL–PML evaluable and non-evaluable specimens

**Variable**	**TEL–PML evaluable**	**TEL–PML non-evaluable**	**p-value**
Age at diagnosis, years	20.5 (15.8–31.2)	18.5 (14.0–25.0)	0.090
Female sex	18/45 (40.0%)	28/52 (53.8%)	0.222
Smoking	6/44 (13.6%)	7/50 (14.0%)	1.000
Amputation specimen	28/39 (71.8%)	22/49 (44.9%)	0.017
Non-osteoblastic subtype	28/45 (62.2%)	41/52 (78.8%)	0.078
Neoadjuvant chemotherapy	30/41 (73.2%)	36/48 (75.0%)	1.000
Adjuvant chemotherapy	37/40 (92.5%)	43/49 (87.8%)	0.506
Radiotherapy	1/21 (4.8%)	4/27 (14.8%)	0.369
Metastasis during follow-up	30/42 (71.4%)	36/52 (69.2%)	1.000
Recurrence	5/45 (11.1%)	6/52 (11.5%)	1.000
Death at last follow-up	29/44 (65.9%)	33/51 (64.7%)	1.000

Continuous variables are shown as median (IQR) and compared with the Mann–Whitney U test. Categorical variables are shown as n/N (%) and compared with Fisher's exact test. The main difference was a higher proportion of amputation specimens among evaluable cases.
