# Supplementary material for: Telomeric DNA–Promyelocytic Leukemia (TEL–PML) Colocalization as an ALT Proxy in Relation to Metastatic Behavior in Osteosarcoma: A Retrospective Cohort Study
Source: Curr Issues Mol Biol. 2026 May 25;48(6):553. doi: 10.3390/cimb48060553 (PMC13297514; doi:10.3390/cimb48060553)
Supplement: Supplementary file 1 [file cimb-48-00553-s001.zip › Table S2.pdf]

Table S2. Treatment-adjusted sensitivity analyses for the association between amputation and TEL–PML evaluability.

Model	N	OR	95% CI	p-value
Adjusted for age, sex, smoking, and initial treatment category	80	4.57	1.57–13.30	0.005
Adjusted for age, sex, smoking, and neoadjuvant chemotherapy	81	4.19	1.52–11.56	0.006
Adjusted for age, sex, smoking, and adjuvant chemotherapy	80	3.49	1.23–9.87	0.019
Adjusted for age, sex, smoking, and radiotherapy	47	2.92	0.81–10.57	0.103

Each estimate corresponds to a separate logistic regression model with TEL–PML evaluability (evaluable vs non-evaluable) as the outcome and amputation as the predictor of interest. Models were additionally adjusted for age, sex, smoking, and one treatment-related variable available in the dataset. N reflects complete-case data for each model. OR odds ratio; CI, confidence interval
