# Supplementary material for: Telomeric DNA–Promyelocytic Leukemia (TEL–PML) Colocalization as an ALT Proxy in Relation to Metastatic Behavior in Osteosarcoma: A Retrospective Cohort Study
Source: Curr Issues Mol Biol. 2026 May 25;48(6):553. doi: 10.3390/cimb48060553 (PMC13297514; doi:10.3390/cimb48060553)
Supplement: Supplementary file 1 [file cimb-48-00553-s001.zip › Table S3.pdf]

Table S3. Firth penalized sensitivity analysis for TEL–PML evaluability

Model	N	OR	95% CI	p-value
Firth adjusted for age, sex, smoking, and initial treatment category	80	3.86	1.40–10.64	0.003
Firth adjusted for age, sex, smoking, and neoadjuvant chemotherapy	81	3.69	1.40–9.75	0.003
Firth adjusted for age, sex, smoking, and adjuvant chemotherapy	80	3.13	1.15–8.49	0.010
Firth adjusted for age, sex, smoking, and radiotherapy	47	2.49	0.75–8.29	0.075

Outcome: TEL–PML evaluability (evaluable vs non-evaluable). Predictor of interest: amputation. Models were adjusted as indicated. These analyses complement the conventional logistic regression sensitivity analyses.
