# Supplementary material for: Telomeric DNA–Promyelocytic Leukemia (TEL–PML) Colocalization as an ALT Proxy in Relation to Metastatic Behavior in Osteosarcoma: A Retrospective Cohort Study
Source: Curr Issues Mol Biol. 2026 May 25;48(6):553. doi: 10.3390/cimb48060553 (PMC13297514; doi:10.3390/cimb48060553)
Supplement: Supplementary file 1 [file cimb-48-00553-s001.zip › Table S4.pdf]

Table S4. Covariate-adjusted logistic regression models for TEL–PML and TERT (remaining non-significant associations)

A) Outcome: TEL–PML positivity among evaluable cases only (positive vs negative)

**Predictor**	**N**	**OR**	**OR 95% CI**	***p*-value**
Pathological diagnosis	44	0.79	0.60–1.05	0.102
Pain (1 vs 0)	43	0.09	<0.01–1.34	0.081
Bone enlargement deformity (1 vs 0)	43	2.84	0.47–17.01	0.254
Edema (1 vs 0)	43	NE	NE	0.999
Weight loss (1 vs 0)	43	1.09	0.18–6.71	0.926
Limitation of movement (1 vs 0)	43	0.58	0.06–6.17	0.655
Paresthesia (1 vs 0)	43	NE	NE	0.999
Decreased strength (1 vs 0)	43	NE	NE	0.999
Fracture (1 vs 0)	43	4.78	0.25–92.67	0.301
History of trauma (1 vs 0)	43	0.55	0.11–2.74	0.468
Time between diagnosis and treatment: 1.0 vs 2.0	41	1.55	0.14–17.34	0.722
Time between diagnosis and treatment: 3.0 vs 2.0	41	2.10	0.13–34.26	0.602
Time between diagnosis and treatment: Other vs 2.0	41	4.79	0.62–36.71	0.132
Adjuvant therapy (1 vs 0)	40	NE	NE	0.999
Neoadjuvant therapy (1 vs 0)	41	0.34	0.05–2.10	0.246
Radiotherapy (1 vs 0)	21	NE	NE	0.999
Recurrence (1 vs 0)	44	0.74	0.06–8.56	0.807
Treatment complications (1 vs 0)	10	1.37	<0.01–266.13	0.908
Metastasis (1 vs 0)	42	1.15	0.19–7.02	0.883

Time from surgery to metastasis	26	1.01	0.90–1.13	0.929
Tumor location: right distal femur vs Other	44	1.75	0.26–11.55	0.563
Tumor location: left distal femur vs Other	44	2.00	0.25–15.91	0.511
Socioeconomic level: Indigent vs Socioeconomic level 1	44	NE	NE	0.999
Socioeconomic level: Other vs Socioeconomic level 1	44	NE	NE	0.999
Socioeconomic level: Socioeconomic level 2 vs Socioeconomic level 1	44	0.61	0.12–3.18	0.557
Initial treatment: Other vs Chemotherapy	38	NE	NE	0.999
Initial treatment: Surgery vs Chemotherapy	38	2.05	0.31–13.78	0.458
Current status: Alive vs Deceased	44	0.53	0.09–3.02	0.473
TERT positive (1 vs 0)	39	1.29	0.25–6.76	0.760

B) Outcome: TERT positive (IHC score ≥1 vs 0)

**Predictor**	**N**	OR	**OR 95% CI**	**p-value**
Pathological diagnosis	57	1.10	0.90–1.34	0.344
Pain (1 vs 0)	57	0.66	0.10–4.42	0.671
Bone enlargement deformity (1 vs 0)	57	0.78	0.24–2.58	0.685
Edema (1 vs 0)	57	NE	NE	0.999
Weight loss (1 vs 0)	57	1.35	0.28–6.58	0.707
Limitation of movement (1 vs 0)	57	0.58	0.14–2.44	0.454

Paresthesia (1 vs 0)	57	NE	NE	0.999
Decreased strength (1 vs 0)	57	NE	NE	0.999
Fracture (1 vs 0)	57	0.94	0.05–16.28	0.964
History of trauma (1 vs 0)	57	0.53	0.16–1.72	0.292
Time between diagnosis and treatment: 1.0 vs 2.0	53	1.66	0.30–9.05	0.561
Time between diagnosis and treatment: 3.0 vs 2.0	53	1.36	0.17–10.72	0.771
Time between diagnosis and treatment: Other vs 2.0	53	0.82	0.16–4.35	0.818
Adjuvant therapy (1 vs 0)	53	4.35	0.40–46.90	0.226
Neoadjuvant therapy (1 vs 0)	53	2.22	0.54–9.18	0.272
Radiotherapy (1 vs 0)	28	0.38	0.04–3.77	0.407
Recurrence (1 vs 0)	57	0.43	0.09–1.97	0.276
Treatment complications (1 vs 0)	17	NE	NE	0.999
Metastasis (1 vs 0)	55	0.98	0.25–3.78	0.978
Time from surgery to metastasis	34	1.05	0.96–1.16	0.272
Tumor location: right distal femur vs Other	57	0.35	0.07–1.65	0.183
Tumor location: left distal femur vs Other	57	2.77	0.47–16.26	0.259
Tumor location: right proximal tibia vs Other	57	4.34	0.35–54.76	0.256
Socioeconomic level: Indigent vs Socioeconomic level 1	57	1.75	0.29–10.67	0.543
Socioeconomic level: Socioeconomic level 2 vs Socioeconomic level 1	57	0.96	0.26–3.58	0.947

Socioeconomic level: Socioeconomic level 3 vs Socioeconomic level 1	57	3.64	0.30–44.57	0.312
Initial treatment: Other vs Chemotherapy	51	0.67	0.05–9.47	0.767
Initial treatment: Surgery vs Chemotherapy	51	0.41	0.11–1.59	0.198
Current status: Alive vs Deceased	57	2.70	0.65–11.25	0.174
TEL-PML positive (1 vs 0)	39	1.24	0.25–6.17	0.789
TEL-PML colocalization: Necrosis (non-evaluable) vs Negative	57	3.10	0.78–12.29	0.108

ORs and 95% CIs were obtained by exponentiating β coefficients and their corresponding confidence intervals from the original adjusted logistic regression models. Rows with quasi-complete separation or extremely unstable estimates are shown as NE (not estimable for clinically meaningful interpretation). Each estimate corresponds to a separate covariate-adjusted logistic regression model including one predictor of interest plus age, sex, and smoking.
