# Supplementary material for: Telomeric DNA–Promyelocytic Leukemia (TEL–PML) Colocalization as an ALT Proxy in Relation to Metastatic Behavior in Osteosarcoma: A Retrospective Cohort Study
Source: Curr Issues Mol Biol. 2026 May 25;48(6):553. doi: 10.3390/cimb48060553 (PMC13297514; doi:10.3390/cimb48060553)
Supplement: Supplementary file 1 [file cimb-48-00553-s001.zip › Table S5.pdf]

Table S5. Clinical outcomes by TEL-PML colocalization status (evaluable cases).

Outcome	TEL-PML positive (n=10)	TEL-PML negative (n=35)	*p*-value
Metastasis (METS = 1)	6/8 (75.0%)	24/34 (70.6%)	1.000
Time to metastasis (months), median (IQR)†	15 (9.8-22.5)	12.5 (10.2-24)	0.926
Early metastasis (<= 6 months) among cases with recorded timing	0/6 (0.0%)	3/20 (15.0%)	1.000
Recurrence (Recurrence = 1)	1/10 (10.0%)	4/35 (11.4%)	1.000
Deceased at last follow-up (Current status category = 2)	7/9 (77.8%)	22/35 (62.9%)	0.695

Categorical outcomes compared using Fisher's exact test; time to metastasis compared using the Mann-Whitney U test. Time-to-metastasis and early-metastasis analyses were restricted to metastatic cases with recorded Tiempo_SX-METS. Early metastasis was defined as Tiempo_SX-METS <= 6 months. Univariable odds ratio (OR) for metastasis: 1.25 (95% CI 0.21-7.28). Denominators vary across rows according to outcome-specific data availability.
