# Supplementary material for: Telomeric DNA–Promyelocytic Leukemia (TEL–PML) Colocalization as an ALT Proxy in Relation to Metastatic Behavior in Osteosarcoma: A Retrospective Cohort Study
Source: Curr Issues Mol Biol. 2026 May 25;48(6):553. doi: 10.3390/cimb48060553 (PMC13297514; doi:10.3390/cimb48060553)
Supplement: Supplementary file 1 [file cimb-48-00553-s001.zip › Table S6.pdf]

Table S6. Firth penalized logistic regression for clinical outcomes according to TEL–PML positivity among evaluable cases.

Outcome	N	Events in TEL–PML positive	Events in TEL–PML negative	Firth OR	95% CI	p-value
Metastasis during follow-up	42	6/8 (75.0%)	24/34 (70.6%)	1.11	0.23–6.86	0.896
Recurrence	45	1/10 (10.0%)	4/35 (11.4%)	1.11	0.10–7.02	0.922
Death at last follow-up	44	7/9 (77.8%)	22/35 (62.9%)	1.80	0.40–10.75	0.454
Early metastasis ≤6 months among cases with recorded timing	26	0/6 (0.0%)	3/20 (15.0%)	0.38	0.003–4.85	0.507

Models were fitted as univariable Firth penalized logistic regressions with each clinical outcome as the dependent variable and TEL–PML positivity (positive vs negative) as the predictor of interest. Analyses were restricted to TEL–PML-evaluable cases. Odds ratios greater than 1 indicate higher odds of the outcome among TEL–PML-positive tumors. Confidence intervals and p values were obtained from profile penalized likelihood. Early metastasis was defined as Tiempo_SX–METS ≤6 months and was evaluated only among cases with recorded timing data. Denominators vary according to outcome-specific data availability. OR, odds ratio; CI, confidence interval; TEL–PML, telomeric DNA–promyelocytic leukemia.
