# Supplementary material for: Telomeric DNA–Promyelocytic Leukemia (TEL–PML) Colocalization as an ALT Proxy in Relation to Metastatic Behavior in Osteosarcoma: A Retrospective Cohort Study
Source: Curr Issues Mol Biol. 2026 May 25;48(6):553. doi: 10.3390/cimb48060553 (PMC13297514; doi:10.3390/cimb48060553)
Supplement: Supplementary file 1 [file cimb-48-00553-s001.zip › Table S7.pdf]

Table S7. Inverse probability weighting sensitivity analysis for clinical outcomes according to TEL–PML positivity among evaluable cases.

Outcome	N	TEL–PML positive events	TEL–PML negative events	IPW-weighted event probability: TEL–PML positive	IPW-weighted event probability: TEL–PML negative	IPW-weighted OR	95% CI	p-value
Metastasis during follow-up	42	6/8	24/34	75.1%	68.8%	1.37	0.19–9.84	0.755
Recurrence	45	1/10	4/35	7.9%	10.3%	0.74	0.04–12.90	0.837
Death at last follow-up	44	7/9	22/35	77.6%	65.3%	1.85	0.27–12.75	0.534
Early metastasis (≤6 months) among cases with recorded timing	26	0/6	3/20	0.0%	11.9%	NE	NE	NE

Stabilized inverse probability weights were estimated from a logistic model for TEL–PML evaluability using age, sex, smoking, amputation specimen, non-osteoblastic histologic subtype, and missing-indicator terms for smoking and amputation. IPW-weighted logistic regression models were restricted to TEL–PML-evaluable cases with available outcome data and used TEL–PML positivity as the predictor of interest. Early metastasis was defined as time from surgery to metastasis ≤6 months among cases with recorded timing data; the weighted OR was not estimated for this outcome because there were no early metastatic events among TEL–PML-positive cases. OR, odds ratio; CI, confidence interval; IPW, inverse probability weighting; NE, not estimable.
