# Supplementary material for: Telomeric DNA–Promyelocytic Leukemia (TEL–PML) Colocalization as an ALT Proxy in Relation to Metastatic Behavior in Osteosarcoma: A Retrospective Cohort Study
Source: Curr Issues Mol Biol. 2026 May 25;48(6):553. doi: 10.3390/cimb48060553 (PMC13297514; doi:10.3390/cimb48060553)
Supplement: Supplementary file 1 [file cimb-48-00553-s001.zip › Table S8.pdf]

Table S8. Clinical outcomes by TERT immunohistochemistry status (scorable cases).

Outcome	TERT positive (n=33)	TERT negative (n=25)	*p*-value
Metastasis (METS = 1)	24/31 (77.4%)	18/24 (75.0%)	1.000
Time to metastasis (months), median (IQR)	24 (9.2-24)	14 (11-24)	0.635
Early metastasis (<= 6 months) among timed cases	2/18 (11.1%)	2/16 (12.5%)	1.000
Recurrence (yes)	4/33 (12.1%)	5/25 (20.0%)	0.479
Deceased at last follow-up (current status category = 2)	22/33 (66.7%)	20/24 (83.3%)	0.226

TERT positive was defined as immunohistochemistry score ≥1, and TERT negative as score 0. Analyses were restricted to cases with scorable TERT immunohistochemistry (n=58). Denominators vary across rows according to outcome-specific data availability. Categorical outcomes were compared using Fisher's exact test, and time to metastasis was compared using the Mann–Whitney U test. Time-to-metastasis and early-metastasis analyses were restricted to metastatic cases with recorded Tiempo_SX–METS. Early metastasis was defined as Tiempo_SX–METS ≤6 months. Univariable odds ratio (OR) for metastasis: 1.14 (95% CI 0.33–3.99).
